# Evaluation of an Infrapopliteal Drug-Eluting Resorbable Scaffold: Design Methodology for the LIFE-BTK Randomized Controlled Trial

**DOI:** 10.1016/j.jscai.2023.100964

**Published:** 2023-05-19

**Authors:** Ramon L. Varcoe, Sahil A. Parikh, Brian G. DeRubertis, Jennifer M. Jones-McMeans, Nutte Tarn Teraphongphom, Jin Wang, Raghu Kolluri, Ido Weinberg, Andrew H. Holden, Hector M. Garcia-Garcia, Steven W.C. Kum, Marc P. Bonaca, Danielle R. Bajakian, Lawrence A. Garcia, Prakash Krishnan, Ehrin Armstrong, Mehdi H. Shishehbor, John Rundback, D. Chris Metzger

**Affiliations:** a The Prince of Wales Hospital, University of New South Wales, Randwick, New South Wales, Australia; b Columbia University Vagelos College of Physicians and Surgeons, New York, New York; c NewYork-Presbyterian/Weill Cornell Medical Center, New York, New York; d Abbott Vascular, Santa Clara, California; e Syntropic Core Lab, Columbus, Ohio; f VasCore, Boston, Massachusetts; g Auckland Hospital, University of Auckland, Grafton, Auckland, New Zealand; h MedStar Washington Hospital Center, Washington, District of Columbia; i Department of Surgery, Changi General Hospital, Singapore; j Cardiovascular Division, CPC Clinical Research, University of Colorado School of Medicine, Aurora, Colorado; k Vascular Care Group, Tufts University School of Medicine, Boston, Massachusetts; l The Mount Sinai Hospital, New York, New York; m Advanced Heart and Vein Center, Denver, Colorado; n University Hospitals Harrington Heart and Vascular Institute, Cleveland, Ohio; o Advanced Interventional and Vascular Services LLP, Teaneck, New Jersey; p Ballad Health, Kingsport, Tennessee

**Keywords:** absorbable implants, angioplasty, arterial occlusive diseases, ischemia, lower extremity, peripheral arterial disease, polymers, stents

## Abstract

**Background:**

Critical limb-threatening ischemia (CLTI) is a severe condition characterized by rest pain and ischemic tissue loss that affects 5% to 10% of people with peripheral artery disease. In the United States, there are few Food and Drug Administration-approved devices for the primary treatment of arteries below-the-knee (BTK). Unfortunately, all suffer from high restenosis rates due to intimal hyperplasia, elastic recoil, and untreated dissection because of a lack of scaffolding. The Esprit BTK system is a resorbable, drug-eluting scaffold device with the potential to address an unmet need in people suffering from CLTI because of infrapopliteal atherosclerosis. The LIFE-BTK (pivotaL Investigation of saFety and Efficacy of drug-eluting resorbable scaffold treatment-Below The Knee) randomized controlled trial (RCT) is a prospectively designed premarket evaluation of the Esprit BTK drug-eluting resorbable scaffold used in the treatment of those patients.

**Methods:**

The LIFE-BTK trial enrolled 261 subjects with CLTI for the RCT and a further 7 subjects for a pharmacokinetic substudy. The objective of the RCT was to evaluate the safety and efficacy of the Esprit BTK scaffold compared to percutaneous transluminal angioplasty. The primary efficacy end point was a composite of limb salvage and primary patency at 12 months. The primary safety end point is freedom from major adverse limb events and peri-operative death at 6 months and 30 days, respectively. Clinical follow-up care is planned for 5 years.

**Conclusions:**

Novel devices must be tested in RCTs to evaluate their safety and efficacy compared to the standard of care if we are to improve outcomes for this challenging group of patients.

## Highlights

• Infrapopliteal interventions are still limited by durability because of restenosis and acute recoil.
• Evidence suggests drug-eluting resorbable scaffolds may improve mid-long-term patency.
• A randomized controlled trial comparing the Esprit scaffold to percutaneous angioplasty.
• The trial design methodology is presented in detail.

## Central Illustration

**Figure:**
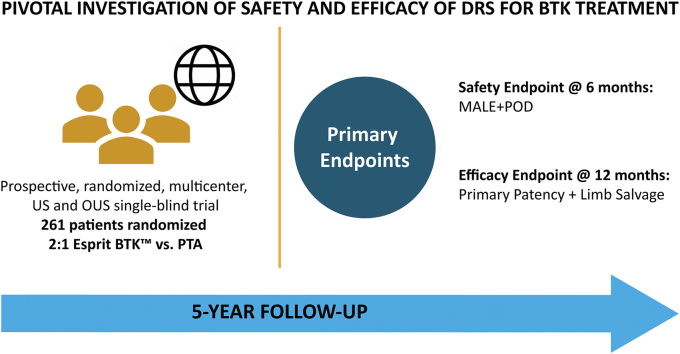

## Introduction

Contemporary percutaneous interventions for occlusive arterial disease of the infrapopliteal circulation enjoy high technical success rates. Advances in guidewire, balloon and crossing catheter technology, pedal access, and bidirectional wire techniques have transformed this interventional space. However, treatment with percutaneous transluminal angioplasty (PTA) still suffers from high rates of elastic recoil, dissection, and short- to mid-term restenosis, as crural arteries are small in caliber and typically carry a high burden of atherosclerotic disease. Treatment of tibial arteries with percutaneous atherectomy may reduce the atherosclerotic burden and decrease the propensity for dissection, but restenosis remains common with this heterogenous class of devices, and distal embolization is a concern for many interventionalists.

Much work has been done to achieve greater durability through the development of new treatment options; however, the field is littered with trials that have failed to show a patency advantage over PTA alone.^1^, ^2^, ^3^, ^4^ Drug-coated balloons, which deposit antiproliferative agents into the blood vessel wall to prevent neointimal proliferation, have been demonstrated effective in arteries above the knee.^5^, ^6^, ^7^, ^8^, ^9^, ^10^, ^11^ However, very few randomized controlled trials (RCTs) have shown an advantage in the infrapopliteal circulation, and there remain no FDA-approved drug-coated balloons for the below-knee arterial circulation.^12^^,^^13^ This may be because the typical lesions in this region are long, heavily calcified, chronic total occlusions prone to mechanical failure from postangioplasty recoil and flow-limiting dissection. Coronary drug-eluting stents have shown promise,^14^, ^15^, ^16^, ^17^ but they have drawbacks that limit their widespread adoption. They are too short to be practically applied to longer lesions; they may act as an impediment to future surgical or endovascular intervention and produce artifacts on cross-sectional imaging such as computed tomography and magnetic resonance imaging. A drug-eluting, resorbable scaffold (DRS) has several advantages, which make it well-suited to the infrapopliteal circulation, and some have demonstrated promising early results.^18^, ^19^, ^20^, ^21^, ^22^ These devices have the properties of a stent which allow them to overcome mechanical modes of failure, and they act as a delivery platform for the antiproliferative drug during the restenotic phase after the percutaneous intervention. Finally, these devices undergo resorption when no longer required, a process that facilitates remodeling of the diseased blood vessel wall.

The goal of this manuscript is to describe the design of the LIFE-BTK (pivotaL Investigation of saFety and Efficacy of drug-eluting resorbable scaffold treatment-Below The Knee) RCT, which is a large multicenter study whose objectives were to evaluate the safety and efficacy of the new everolimus-coated DRS Esprit BTK (Abbott Vascular) for the treatment of diseased infrapopliteal lesions in subjects with critical limb-threatening ischemia (CLTI). Included is a substudy designed to determine the pharmacokinetics (PKs) of everolimus delivered by the scaffold in a separate nonrandomized cohort.

### Esprit BTK DRS

**Figure 1:**
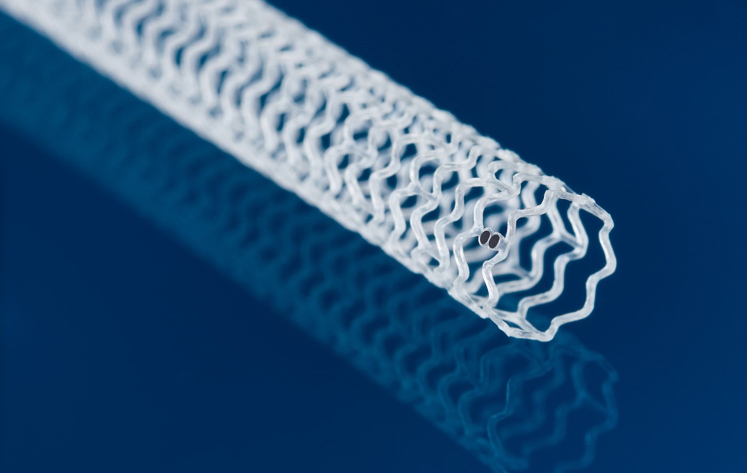
**The Esprit BTK drug-eluting resorbable scaffold.** BTK, below-the-knee.

The novel Esprit BTK vascular scaffold (Figure 1) consists of a poly-L-Lactic acid (PLLA) structure coated with a 7 μm poly-D,L,lactide (PDLLA) surface polymer that controls the release of the antiproliferative drug everolimus at a concentration of 100 μg/mm^2^. Both structure and polymer are fully biodegradable. The drug dose density and elution profile are identical to that of the XIENCE drug-eluting stents (Abbott Vascular). The long chains of PDLLA and PLLA are progressively shortened as ester bonds between lactide repeat units are hydrolyzed, and toward the end of the resorption process, small particles <2 μm are phagocytosed by macrophages. Ultimately both PLLA and PDLLA degrade to lactic acid and are metabolized through the Krebs cycle to form carbon dioxide and water. The current generation Esprit BTK DRS design consists of circumferential hoops connected to one another by straight bridges. The Esprit BTK scaffold comes in larger diameters and longer lengths and has thinner struts than the coronary predicate device. Its struts are 99 to 120 μm thick, depending on the scaffold diameter. The scaffold lengths available during the trial were 18, 28, and 38 mm, with 2.5, 3.0, 3.5, and 3.75 mm diameters, which could be safely postdilated 0.5 mm beyond their nominal diameter.

## Materials and methods

### Study design

The LIFE-BTK trial is a prospective, multicenter, single-blind RCT designed to support the United States premarket approval of the Esprit BTK system (Central Illustration). The LIFE-BTK study is registered at ClinicalTrials.gov (unique identifier: NCT04227899). Subjects were randomized in a 2:1 ratio to the Esprit BTK and PTA arms of the study.

**Central Illustration:**
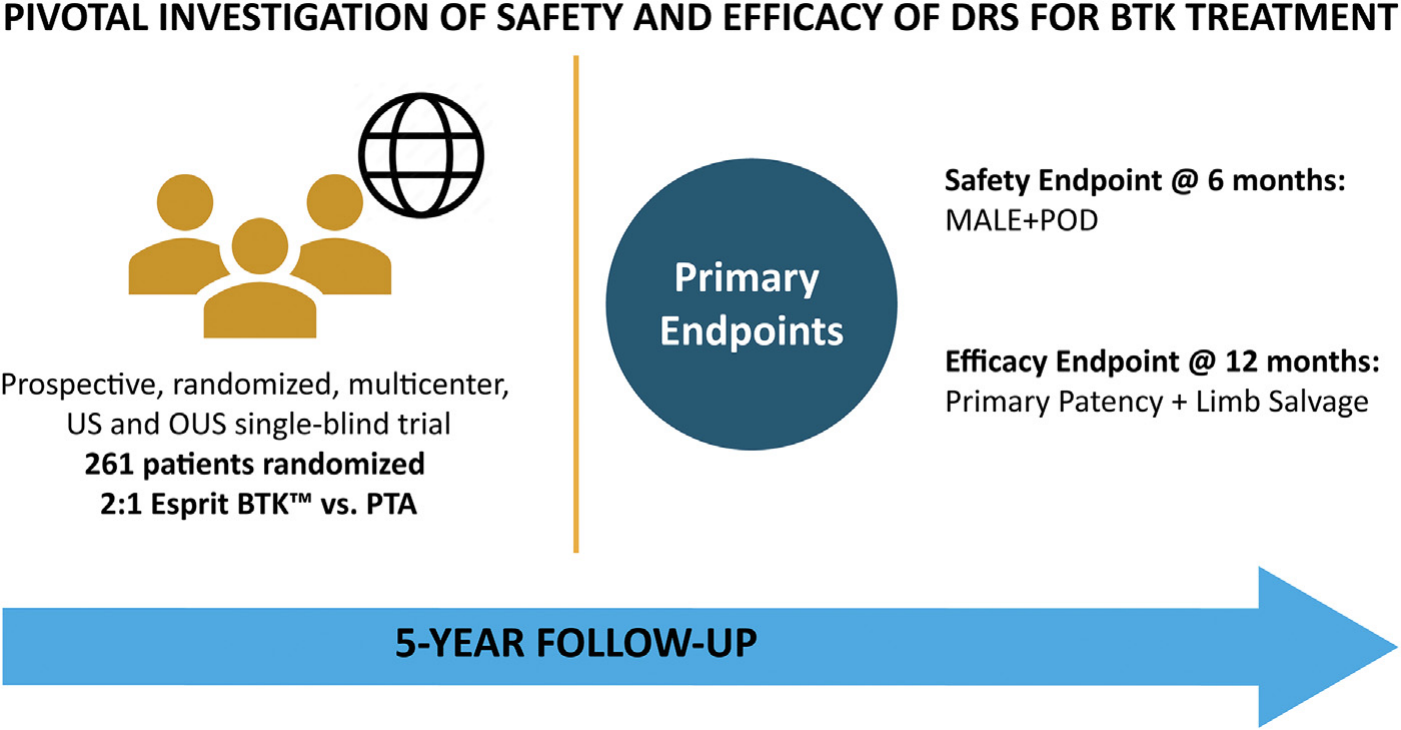
Design of the LIFE-BTK randomized controlled trial.

Core laboratories were utilized to adjudicate angiography, intravascular ultrasound, and optical coherence tomography (MedStar Health), as well as duplex ultrasound (DUS) (VasCore) and quantitative wound assessment (Syntropic CoreLab).

### Study population and eligibility

LIFE-BTK RCT was a multicenter (52 sites) study that enrolled subjects across 6 countries that included the United States, Singapore, Hong Kong, Taiwan, Australia, and New Zealand. Prior to enrollment, all subjects underwent a rigorous informed consent process guided by applicable regulations from the center’s institutional review board or ethics committee.

Subjects with arterial narrowing in infrapopliteal lesions causing CLTI, Rutherford-Becker class 4 or 5, were eligible for inclusion. The full list of LIFE-BTK inclusion and exclusion criteria is given in Table 1. Subject randomization occurred after all eligibility criteria were met, all inflow and nontarget lesion(s) had been treated successfully, and the guidewire had successfully crossed the target lesion.

**Table 1:** Inclusion and exclusion criteria for the LIFE-BTK randomized controlled trial and pharmacokinetic substudy.

LIFE-BTK RCT	LIFE-BTK PK substudy
Inclusion criteria	

General inclusion criteria	

1) Subject must provide written informed consent prior to any clinical investigation-related procedure.	1) Same as RCT
2) Subject has symptomatic critical limb-threatening ischemia, Rutherford-Becker clinical category 4 or 5.	2) Same as RCT
3) Subject requires primary treatment of up to 2 de novo or restenotic (treated with prior PTA) infrapopliteal lesions.	3) Subject requires primary treatment of 1 or more de novo or restenotic (treated with prior PTA) infrapopliteal lesions.
4) Subject must be at least 18 y of age.	4) Same as RCT
5) Female subjects of childbearing potential should not be pregnant and must be on birth control.	5) Same as RCT

Anatomic inclusion criteria	

1) Up to 2 native infrapopliteal lesions, each lesion located in a separate infrapopliteal vessel in the same limb. Restenotic (from prior PTA) lesions are allowed. a) Lesion must be located in the proximal 2/3 of native infrapopliteal vessels, with vessel diameter of ≥2.5 mm and ≤4.00 mm by investigator visual assessment. b) Total scaffold length to completely cover/treat a target lesion must not exceed 170 mm (total everolimus drug dose of 1790 μg). c) The total scaffold length among all target lesions must not exceed 170 mm. d) The target vessel cannot have any other angiographic significant lesions (≥50%). e) Tandem lesions are allowed if they are <3 cm apart, and the total scaffold length used to cover the entire diseased segment is ≤170 mm. Each tandem lesion is considered 1 lesion.	1) One or more native infrapopliteal lesions, including de novo lesions and restenotic (from prior PTA) lesions, are allowed. a) Same as RCT b) Total scaffold length to completely cover/treat target lesion(s) must be between 170 mm and 256 mm (maximum total everolimus drug dose of 2714 μg). c) The target vessel can have other angiographic significant lesions (≥50%) that should be treated per the institution’s standard of care prior to treatment of the target lesion. d) Tandem lesions are allowed, and the total scaffold length used to cover the entire diseased segment must be ≤256 mm.
2) Target lesion(s) must have ≥70% stenosis per visual assessment at the time of the procedure. If needed, quantitative imaging (angiography, intravascular ultrasound, and/or optical coherence tomography) can be used to aid the accurate sizing of the vessels.	2) Same as RCT
3) The distal margin of the target lesion must be located ≥10 cm proximal to the proximal margin of the ankle mortise. The vessel segment distal to the target lesion must be patent all the way to the ankle, with no significant lesion (≥50% stenosis).	3) The distal margin of the scaffold must be located ≥10 cm proximal to the proximal margin of the ankle mortise. If the vessel segment distal to the target lesion has a significant lesion (>50% stenosis), it should be treated per the institution’s standard of care prior to deployment of the scaffold.
4) Significant lesions (≥50% stenosis) in the inflow artery(ies) must be treated successfully (as per the physician's assessment of the angiography) through the standard of care prior to the treatment of the target lesion. Treatment can be done within the same trial procedure.	4) Significant lesions (≥50% stenosis) in the inflow artery(ies) must be treated successfully (as per the physician’s assessment of the angiography) through the standard of care prior to the treatment of the target lesion. Treatment must be done within the same trial procedure. Treatment allowed for inflow artery lesions is PTA, atherectomy, cutting/scoring balloon, shockwave balloon, bare metal stent, drug-eluting stents, or drug-coated balloon. Everolimus-coated or eluting devices are not allowed.
5) Nontarget lesion(s) (if applicable) must be located in separate infrapopliteal vessel(s) from the target lesion and suitable to be treated per the institution’s standard of care.	5) It is acceptable for nontarget lesion(s) (if applicable) to be located in the same infrapopliteal vessel(s) as the target lesion and suitable to be treated per the institution’s standard of care. Nontarget lesions must be treated successfully prior to target lesions and not require recross of the scaffold.
6) Guidewire must cross the target lesion successfully. Crossing in an antegrade fashion is preferred, but retrograde crossing may be used. However, the treatment must be delivered antegrade.	6) Same as RCT

Exclusion criteria	

General exclusion criteria	

1) Subject is currently participating in another clinical investigation that has not yet completed its primary end point.	Same as RCT
2) Pregnant or nursing subjects and those who plan pregnancy during the clinical investigation follow-up period.	
3) Presence of other anatomic or comorbid conditions or other medical, social, or psychological conditions that, in the investigator's opinion, could limit the subject's ability to participate in the clinical investigation or to comply with follow-up requirements.	
4) Incapacitated individuals, defined as persons who are mentally ill, mentally handicapped, or individuals without legal authority, are excluded from the study population.	
5) Subject has had any amputation to the ipsilateral extremity other than the toe or forefoot, or the subject has had a major amputation to the contralateral extremity <1 y prior to the index procedure and is not independently ambulating.	
6) Subject has known hypersensitivity or contraindication to device material and its degradants (everolimus, poly [L-lactide], poly [DL-lactide], lactide, lactic acid) and cobalt, chromium, nickel, platinum, tungsten, acrylic, and fluoro polymers that cannot be adequately premedicated. Subject has a known contrast sensitivity that cannot be adequately premedicated.	
7) Subject has known allergic reaction, hypersensitivity, or contraindication to aspirin; or to adenosine-diphosphate antagonists, such clopidogrel, prasugrel, or ticagrelor; or to anticoagulants such as heparin or bivalirudin, and therefore cannot be adequately treated with study medications. Subject with planned surgery or procedure necessitating discontinuation of antiplatelet medications within 12 mo after the index procedure. Planned amputation that will necessitate discontinuation of antiplatelet medications is allowed.	
8) Subject has a life expectancy of ≤1 y.	
9) Subject has had a stroke within the previous 3 mo with a residual Rankin score of ≥2.	
10) Subject has renal insufficiency as defined as an estimated glomerular filtration rate <30 mL/min/1.73 m^2^.	
11) Subject is currently on dialysis.	
12) Subject has platelet count <100,000 cells/mm^3^ or >700,000 cells/mm^3^, a white blood cell (WBC) <3000 cells/mm^3^, or hemoglobin <9.0 g/dL.	
13) Subject has known serious immunosuppressive disease (eg, human immunodeficiency virus), or has a severe autoimmune disease that requires chronic immunosuppressive therapy (eg, systemic lupus erythematosus, etc), or the subject is receiving immunosuppression therapy for other conditions. Subjects treated for HIV and who have an undetectable viral load, such that their immune system is not considered compromised, are eligible.	
14) Subject has a body mass index of <18 kg/m^2^.	
15) Subject is receiving or scheduled to receive anticancer therapy for malignancy within 6 mo prior to the index procedure or within 1 y after the procedure. Patients taking medications classified as chemotherapy but who have been in remission for at least 6 mo are eligible.	
16) Subject has a coagulation disorder that increases the risk of arterial thrombosis. Subjects with deep vein thrombosis and disorders that increase the risk of deep vein thrombosis can be included in the study.	
17) Subject who requires thrombolysis as a primary treatment modality or requires other treatment for acute limb ischemia of the target limb.	
18) Subject has previously had or requires surgical revascularization involving any vessel of the ipsilateral extremity. Prior femoropopliteal or aortobifemoral bypass is allowed. Any bypass to the tibial arteries is not allowed.	
19) Subject has signs or symptoms of advanced limb infection or septicemia (fever >38.5°C, WBC >15,000 cells/mm^3^, hypotension) at the time of assessment. Osteomyelitis of the phalanges or metatarsal heads (as described in exclusion criteria #21a) or cellulitis of the foot amenable to treatment with IV antibiotics at the time of revascularization is acceptable.	
20) Subject is bedridden or unable to walk (with assistance is acceptable). Subjects in a wheelchair who are able to mobilize on their own can be enrolled.	
21) Subject with extensive tissue loss is salvageable only with complex foot reconstruction or nontraditional transmetatarsal amputations. This includes subjects with: a) Osteomyelitis that extends proximally to the metatarsal heads. Osteomyelitis limited to the phalanges or metatarsal heads is acceptable for enrollment. b) Gangrene involves the plantar skin of the forefoot, midfoot, or heel. c) Deep ulcer or large shallow ulcer (>3 cm) involving the plantar skin of the forefoot, midfoot, or heel. d) Full-thickness heel ulcer with/without calcaneal involvement. e) Any wound with calcaneal bone involvement. f) Wounds that are deemed to be neuropathic or nonischemic in nature. g) Wounds that would require flap coverage or complex wound management for large soft tissue defects. h) Full-thickness wounds on the dorsum of the foot with exposed tendon or bone.	
22) Subject is unable or unwilling to provide written consent prior to enrollment.	
23) Subject has active symptoms and/or a positive test result of COVID-19 or other rapidly spreading novel infectious agent within the prior 2 mo.	

Anatomic exclusion criteria	

1) Lesions with severe calcification, in which there is a high likelihood that successful predilatation cannot be achieved.	1) Same as RCT
2) Lesion that has prior metallic stent implant.	2) Same as RCT
3) Significant (≥50% stenosis) lesion in a distal outflow artery that would be perfused by the target vessel and that requires treatment at the time of the index procedure.	3) This criterion was removed for PK sub-study
4) Subject has had or will require treatment in any vessel with an everolimus drug-coated or drug-eluting device <30 d prestudy procedure, or during the index procedure, such that the cumulative (Esprit BTK plus everolimus-eluting device) everolimus drug dose exceeds 1790 μg.	4) Coronary or peripheral artery treated with the everolimus-eluting device during index procedure or within 90 d prior to index procedure.
5) Target or (if applicable) nontarget vessel contains visible thrombus as indicated in the angiographic images.	5) Same as RCT
6) Subject has angiographic evidence of thromboembolism or atheroembolism in the ipsilateral extremity. (Pre- and post-angiographic imaging must confirm the absence of emboli in the distal anatomy.)	6) Same as RCT
7) Unsuccessfully treated proximal inflow-limiting arterial stenosis or inflow-limiting arterial lesions left untreated.	7) Same as RCT
8) No angiographic evidence of a patent pedal artery.	8) Same as RCT
9) Target or (if applicable) nontarget lesion location requiring a bifurcation treatment method that requires scaffolding of both branches (without the intention of scaffolding both branches, provisional treatment is acceptable).	9) Same as RCT
10) Aneurysm in the iliac, common femoral, superficial femoral, popliteal, or target artery of the ipsilateral extremity.	10) Same as RCT
11) Visual assessment of the target lesion suggests that the investigator is unable to predilate the lesion according to the vessel diameter.	11) Same as RCT
12) Target lesion has a high probability that atherectomy will be required at the time of index procedure for the treatment of the target vessel.	12) Same as RCT

IV, intravenous; LIFE-BTK, pivotaL Investigation of saFety and Efficacy of drug-eluting resorbable scaffold treatment-Below The Knee; PK, pharmacokinetic; PTA, percutaneous transluminal angioplasty; RCT, randomized controlled trials.

### Racial diversity in the study population

Peripheral artery disease is common in the community but more so among African American, Hispanic, and Native American races.^23^^,^^24^ In the LIFE-BTK study, strategies were put in place to assist underrepresented populations in gaining access and participating in the clinical trial. A primary focus was to increase the number of diverse physicians, sites, and patients that were participating in the trial. This concept is supported by research demonstrating that patients of diverse backgrounds may find more trust within the health care environment that is similar to them in language, race, and sex. Among the strategies employed for this purpose was the use of the www.LIFE-BTK.com website, patient videos, brochures, and radio media, all of which provided reliable study information and resources to assist investigators in communicating with their patients from diverse backgrounds. Additionally, all patient-facing study materials were phrased in nonmedical lay-person language for potential subjects and their families to better understand the nature of CLTI and clinical trials. Further services were provided to facilitate patient engagement and participation, including translational services that were available 24 hours a day, 7 days a week. Travel and lodging assistance was provided for participants living at a significant distance from the treating facility, and a professional mobile follow-up service was utilized for those who had difficulty returning for scheduled follow-up visits in order to minimize the number of patients lost to follow-up.

## Device implant and trial procedures

### Preprocedure assessments and medications

Routine laboratory assessments, including hemoglobin, hematocrit, white blood count, platelet count, serum creatinine, and blood urea nitrogen, were required <30 days before the index procedure. Subjects were assessed for target limb ischemia status using the Rutherford-Becker classification. Baseline patient-reported outcomes were collected before enrollment. These included overall health status using the EuroQol - 5 Dimensions, 5 Levels tool (EQ-5D-5L), disease-specific health status from the peripheral artery questionnaire (PAQ), and walking performance assessment using the Walking Impairment Questionnaire (WIQ). Ankle-brachial index (ABI) measurement was obtained for the target limb. The toe brachial index was used if ABI could not be reliably measured. All index wounds of the treatment limb were assessed and photographed with a dedicated eKare inSight (eKare, Inc) wound area and volumetric tool according to the wound core laboratory guideline for both volume and etiology.

All patients received a loading dose of ≥300 mg aspirin and a loading dose of P2Y12 receptor inhibitor (clopidogrel, prasugrel, or ticagrelor) between 24 hours prior and no >1 hour after the end of the procedure unless they were already taking these medications regularly, in which case the patient’s standard daily dose was continued. In the Esprit BTK DRS arm, dual antiplatelet therapy was continued for at least 1 year, followed by single-agent therapy thereafter, whereas patients in the PTA arm received dual therapy for 1 month, followed by single-agent therapy thereafter. If patients were on long-term anticoagulation therapy, a single antiplatelet agent was used at the investigator’s discretion, and the anticoagulation therapy was continued.

Angiographic assessment of the inflow, outflow, target, and nontarget lesions was performed to ensure anatomic inclusion criteria were met, with special attention given to vessel diameter for scaffold sizing purposes. The study allowed for the treatment of lesions within arteries of diameter 2.5 to 4.0 mm by visual assessment. An objective method of sizing, such as intravascular ultrasound or optical coherence tomography, was recommended to confirm the visual assessment if there was uncertainty.

### Treatment strategy and technique

Treatment of up to 2 target lesions was allowed in the trial, and these lesions could be in 2 separate tibial arteries. Eligible lesions had to be located in the proximal 2/3 of the lower leg and required a nondiseased run-off vessel to the ankle level beyond the target lesions; thus, no interventions beyond the target lesion were allowed. In addition, restenotic lesions which had previously been treated with PTA could be included. Prior to randomization, all significant lesions of the inflow and nontarget arteries had to be successfully treated without complications, and then once the lesion was successfully crossed, the patient could be randomized. Treatment of the target lesion with any form of atherectomy, scoring/cutting balloon, or intravascular lithotripsy was not permitted in either arm. Patients who were thought to have a high likelihood of benefiting from atherectomy or had severe calcification which might benefit from the use of specialty balloons were excluded from the trial. Planned minor amputation was allowed at the time of the index procedure or within the first month thereafter.

In the Esprit BTK DRS arm, predilatation was mandatory, with noncompliant balloons preferred, as predilatation has been associated with optimal device performance.^25^ Predilatation balloons were sized 1:1 to the reference vessel diameter, and the final scaffold diameter was chosen by using the predilatation balloon size as a guide. Successful predilatation was defined as residual diameter stenosis of <30%.

The length of the DRS was selected to allow at least 2 mm of normal reference vessel at both proximal and distal edges. A maximum total scaffolded length of 170 mm was allowed, and this maximum scaffold length could be divided over up to 2 target lesions. Tandem lesions were allowed if they were <3 cm apart, in which case they were analyzed as a single lesion. Coverage of the entire length of the predilated segment with the implanted DRS was mandated to prevent “geographic miss.” The Esprit BTK scaffold was mounted on a proprietary balloon which was inflated slowly in 2 atm increments every 5 seconds until the scaffold was fully expanded. The pressure was then maintained for 30 seconds to allow the polymer time to expand and hydrate fully. When using multiple Esprit BTK scaffolds, the distal scaffold was deployed first, followed by the proximal scaffold(s), and an abutting, the nonoverlapping configuration, was recommended.

Postdilatation of the scaffold was required for all treated lesions using a noncompliant or semicompliant balloon sized 1:1 to the reference vessel diameter. The postdilatation balloon length was selected such that the balloon stayed within the margins of the scaffold to avoid edge dissection or trauma to the nontreated segment. In addition, the scaffold was not expanded beyond the dilatation limit of 0.5 mm above the nominal diameter to prevent scaffold damage. For the PTA arm, the target lesions were treated per standard of care at the discretion of the proceduralist without mandated inflation times or maximum/minimum inflation pressure to ensure a valid comparison between the Esprit BTK scaffold and contemporary PTA practice. If a PTA balloon used in either study arm was unable to cross or inflate to its nominal diameter, that subject was deregistered.

Successful target lesion treatment was assessed on magnified orthogonal angiographic views of the target lesion, and run-off vessels were assessed to confirm the absence of distal embolization. Acute procedural success was defined as residual diameter stenosis <30%, final number of run-off vessels equivalent to or greater than the number at initial angiography, absence of residual dissection (Grade ≥ type C), and/or angiographic complications including distal embolization, perforation or thrombosis.

## Follow-up

Clinical, in-person follow-up was performed at 30 days, 3 months, 6 months, and 1 year and will be continued annually to complete 5 years of follow-up. These visits assessed adverse events, changes in postprocedure medications, Rutherford-Becker class, and ABI/toe brachial index measurement. Patient-reported outcome measures (EQ-5D-5L, WIQ, and PAQ) were collected for the first year only. For subjects with an index wound, assessments took place to assess healing and infection at 2, 6, and 12 weeks. Quantitative measurements were taken from wound images which were assessed by the wound core laboratory. If the wound was not healed at 3 months, there was an additional assessment at 6- and 12-month follow-ups. New wound occurrence, or recurrence of a previously healed wound, was also assessed and evaluated at ongoing visits. DUS to assess both lesion patency and binary restenosis was performed at 30 days, 6 months, 1, 2, and 3 years, then analyzed by the DUS core laboratory. Table 2 provides a summary of each follow-up visit with assessments performed.

**Table 2:** Summary of follow-up assessments.

Follow-up	Assessments
14 d (±3 d)	Index wound assessment
30 d (±7 d)	Medications review, adverse events review, ABI/TBI measurement, Rutherford-Becker class, index wound assessment, new wound assessment, WIQ, PAQ, EQ-5D-5L
30 d (±14 d)	DUS
42 d (±7 d)	Index wound assessment
90 d (±14 d)	Medications review, adverse events review, ABI/TBI measurement, Rutherford-Becker class, index wound assessment, new wound assessment, WIQ, PAQ, EQ-5D-5L
180 d (±28 d)	DUS, medications review, adverse events review, ABI/TBI measurement, Rutherford-Becker class, index wound assessment, new wound assessment, WIQ, PAQ, EQ-5D-5L
1 y (±28 d)	DUS, medications review, adverse events review, ABI/TBI measurement, Rutherford-Becker class, index wound assessment, new wound assessment, WIQ, PAQ, EQ-5D-5L
2 y (±28 d)	DUS, medications review, adverse events review, ABI/TBI measurement, Rutherford-Becker class, new wound assessment
3 y (±28 d)	DUS, medications review, adverse events review, ABI/TBI measurement, Rutherford-Becker class, new wound assessment
4 y (±28 d)	Medications review, adverse events review, ABI/TBI measurement, Rutherford-Becker class, new wound assessment
5 y (±28 d)	Medications review, adverse events review, ABI/TBI measurement, Rutherford-Becker class, new wound assessment

ABI, Ankle-brachial index; DUS, duplex ultrasound; EQ-5D-5L, EuroQol - 5 Dimensions, 5 Levels; PAQ, peripheral artery questionnaire; TBI, toe brachial index; WIQ, Walking Impairment Questionnaire.

## Trial blinding procedures

All subjects were blinded to their assigned treatment, and study site personnel were trained to avoid disclosing the treatment assignment. Subject blinding was maintained until all subjects had completed their 5-year follow-up visit. The treating physician was not blinded to the assigned treatment. Monitoring source documentation to identify inappropriate unblinding was performed with protocol deviations issued for the patient or unauthorized personnel unblinding.

## End point analysis

### Primary end points

There was a primary efficacy end point and primary safety end point for the RCT.

The primary efficacy end point was a composite of limb salvage and primary patency at 12 months. Specifically, it was defined as freedom from the above-ankle amputation of the index limb, 100% total occlusion of the target vessel, binary restenosis of the target lesion, and clinically-driven target lesion revascularization (CD-TLR). Binary restenosis was defined as the presence of significant restenosis >50% by angiography or peak systolic velocity ratio (PSVR) ≥2.0 by DUS, based on the best available evidence.^26^ Each target lesion was interrogated for the presence of a raised PSVR. The core laboratory then used additional secondary criteria (correlating factors) to confirm any target lesion stenosis. These factors included visible stenosis on B-mode imaging, focal increase in the absolute peak velocity, poststenotic turbulence, change in waveform shape, and/or velocity drop distal to the stenosis. If a PSVR could not be calculated, then these secondary factors were used to determine significant stenosis, or if indeterminate (discordant), the subject was considered nondiagnostic and excluded from the analysis. If a subject underwent both angiogram and DUS at the same time point, the angiogram was used as the primary determinant of binary restenosis.

The primary safety end point was freedom from major adverse limb events and peri-operative death (MALE + POD). MALE included above-ankle amputation of the index limb and major reintervention defined as a new surgical bypass graft, interposition graft, thrombectomy, or thrombolysis. POD was defined as peri-operative mortality from any cause within 30 days of the index procedure.

Both primary analyses must pass for the trial to be successful. PK substudy subjects were not included in the primary analysis population of the LIFE-BTK RCT and will not contribute to determining the primary end points for the RCT.

### Secondary end points

There were 2 statistically powered secondary end points adjudicated at 12 months. These included (1) binary restenosis of the target lesion and (2) a composite end point of freedom from above-ankle amputation of the index limb, total occlusion of the target vessel, and CD-TLR. Details of those power calculations are given in the statistical analysis section.

The complete list of secondary end points is given in Table 3. Some of these are quantitative, such as procedural, technical, and clinical success, amputation-free survival, Rutherford-Becker class, freedom from above-ankle amputation of the index limb, and clinically-driven target lesion/vessel revascularization. Those clinical end points were evaluated at 1 month, 3 months, 6 months, 1 year, and annually through 5 years. Other secondary end points were descriptive, informational end points, which were patient-reported. They include the EQ-5D-5L, WIQ, and PAQ, which are to be analyzed and reported at baseline, 30 days, 3 months, 6 months, and 1 year. Finally, cost per quality-adjusted life-year and cost per clinical event will also be evaluated. The cost analysis will be performed on the prospective data collected as part of the LIFE-BTK study.

**Table 3:** Secondary end points.

Procedural
Acute procedure success
Device success - for Esprit arm only
Technical success
Clinical success
Angiographic acute gain (in-segment)
Angiographic acute gain (in-device) - for Esprit arm only
Clinical end points evaluated at 1 mo, 3 mo, 6 mo, 1 y, and annually through 5 y
Composite of limb salvage and primary patency (primary efficacy end point)
Freedom from MALE+POD (primary safety end point)
Freedom from: above-ankle amputation in index limb, 100% total occlusion of the target vessel, and clinically-driven target lesion revascularization (CD-TLR)
Freedom from major amputation and CD-TLR
Freedom from above-ankle amputation
Freedom from restenosis
Binary restenosis
Amputation-free survival^a^
All-cause death
Arterial thrombosis
Major reintervention on index limb
Primary assisted patency
Secondary patency
CD-TLR
Clinically-driven target vessel revascularization (CD-TVR)
CD-TVR distal to the target lesion
CD-TVR proximal to the target lesion
Index wound assessment for healing (14 d, 30 d, 42 d, 90 d, 180 d, and 1 y)
Index wound assessment for infection (14 d, 30 d, 42 d, 90 d, 180 d, and 1 y)
Rutherford-Becker clinical category, and change from baseline for the treated limb
Occurrence of new wound
Acute limb ischemia
Peripheral embolization

MALE + POD, major adverse limb events and peri-operative death.

a Amputation-free survival is defined as freedom from both above-ankle amputation and death.

## Statistical analysis plan

The primary efficacy end point will be evaluated in a superiority analysis comparing the Esprit BTK DRS arm against PTA at 1 year with a 1-sided α of 0.025 using Pearson’s χ^2^ test or Fisher exact test. An approximate 20% treatment effect favoring the Esprit BTK arm was expected based on historical data.^14^^,^^17^^,^^27^^,^^28^ Our assumption was that the end point rates would be 75% and 55% for the Esprit BTK arm and PTA arm, respectively. Based on those assumptions, an effective sample size of 222 (148 for the Esprit BTK arm and 74 for the PTA arm) would provide approximately 84% power using Pearson’s χ^2^ test or Fisher exact test. Therefore, a sample size of 261 subjects was calculated to account for a 15% attrition rate at 1 year because of the withdrawal, loss to follow-up, and uninterpretable imaging data.

The primary safety end point will be evaluated in a noninferiority analysis using the difference in rates between Esprit BTK and PTA with a 1-sided α of 0.025 calculated with the Farrington-Manning method. It was assumed that the end point rates would be 95% for the Esprit BTK arm and PTA arm, respectively.

In addition, conventional and landmark Kaplan-Meier analyses will be performed for the primary safety and efficacy end points. The landmark analysis will be from 0 to 30 days, 30 days to 6 months, and 30 days to 1 year. The conventional analysis will extend for the entire 5-year follow-up period.

Both powered secondary end points will be evaluated in a superiority analysis using the difference in rates to compare the 2 study arms with a 1-sided α of 0.025. The rates for the first powered secondary end point (12-month binary restenosis) were assumed at 15% and 35% for the Esprit BTK and PTA arms, respectively. For the second powered secondary end point (12-month freedom from index limb major amputation, target vessel occlusion, and CD-TLR), it was assumed that the end point rates would be 83% and 65% for the Esprit BTK and PTA arms, respectively. Those assumptions were based on contemporary published literature.^14^^,^^16^^,^^17^^,^^29^^,^^30^

## PK substudy

The LIFE-BTK PK substudy was a prospective, single-arm, open-label, nonrandomized substudy. It was designed to enroll approximately 7 subjects, all of whom have undergone treatment with the Esprit BTK DRS in narrowed or occluded infrapopliteal arteries. Three of those will have had paclitaxel drug-coated balloon treatment for inflow artery lesions before DRS placement; the other 4 will not.

The objective of the substudy was to determine the PKs of everolimus delivered by the Esprit BTK DRS in a separate, nonrandomized cohort of subjects receiving the Esprit DRS. Eligibility criteria were similar to the RCT, and exceptions included subtle differences in the angiographic inclusion criteria, the number of target lesions, and the total scaffolded segment length allowed. In the substudy, there was no limit on the number of lesions that could be treated, and the total scaffold length must have been between 170 to 256 mm, compared to the maximum 170 mm allowed in the RCT. In addition, the longer-treated segments were included to evaluate the PKs of everolimus at a higher drug dose. The list of PK substudy inclusion and exclusion criteria is also given in Table 1.

The scaffold implantation strategy for the PK substudy was the same as for the RCT. Staged procedures, where an inflow lesion was treated in a separate encounter, were allowed in the RCT but not in the PK substudy.

Eurofins I ADME BIOANALYSES was the core laboratory used for blood sample analysis. They provided materials for blood sampling and handling. In brief, arterial or venous blood samples were collected in ethylenediaminetetraacetic acid-coated tubes, stored, processed, and shipped according to the Investigator Laboratory Manual. Blood samples were taken before, during, and at specific intervals after the procedure (Table 4) to determine the systemic release kinetics of everolimus. The PK analysis of everolimus was then carried out by the core laboratory, which included analysis of PK parameters t_max_, C_max_, AUC_0-24h_, AUC_0-t_, AUC_0-∞_, λ_z_, t_1/2term_, and CL (Table 5). Whole blood concentration-time data was listed by nominal sampling time. To explore the dose proportionality of everolimus, a regression analysis on dose normalized to 1 μg for everolimus was performed.

**Table 4:** Pharmacokinetic substudy blood draw time points.

Time point	Allowed time window
Preprocedure	On the day of the index procedure prior to implantation of the first Esprit BTK
Index procedure blood draw 1	Immediately after the first scaffold has been implanted, this can be arterial from the sheath
Index procedure blood draw 2 and/or more	Every 15 min after the first scaffold has been implanted until the last scaffold has been implanted, this can be arterial from the sheath
0 min	When the last Esprit BTK is deployed, ie, the last Esprit BTK delivery catheter is removed from the body
10 min	±2 min
30 min	±6 min
1 h	±12 min
2 h	±24 min
4 h	±48 min
6 h	±72 min
12 h	±144 min
24 h (1 d)	±4.8 h
48 h (2 d)	±9.6 h
72 h (3 d)	±14.4 h
96 h (4 d)	±19.2 h
120 h (5 d)	±24 h
168 h (7 d)	±33.6 h
336 h (14 d)	±67.2 h
720 h (30 d)	±144 h
1440 h (60 d)	±288 h

BTK, below-the-knee.

**Table 5:** Pharmacokinetic Substudy analysis parameters.

Parameter	Definition
C_max_ (ng/mL)	Maximal observed blood everolimus concentration
t_max_ (h)	Time to reach the maximal observed blood everolimus concentration
AUC_24 h_ (ng∗h/mL)	Area under the blood everolimus concentration vs time curve from time 0 up to 24 h postplacement of the last Esprit BTK, calculated by the linear up/log down trapezoidal method
AUC_last_ (ng∗h/mL)	Area under the blood everolimus concentration vs time curve from time 0 up to the last quantifiable concentration, calculated by the linear up/log down trapezoidal method
AUC_0-∞_ (ng∗h/mL)	Area under the blood everolimus concentration vs time curve from time zero and extrapolated to infinite time,
λ_z_ (1/h)	Terminal rate constant, determined by linear regression of terminal points of the in-linear analyte concentration-time curve
t_1/2_ (h)	Terminal half-life, calculated as t_1/2_ = 0.693/λ_z_
CL (L/h)	Clearance, calculated as dose/AUC_0-∞_

## Conclusion

Fully resorbable, drug-eluting scaffolds have the potential to reduce the intimal hyperplasia and restenosis which plague percutaneous angioplasty. In addition, the scaffolds used in the LIFE-BTK trial provide mechanical support to overcome the elastic recoil, residual plaque burden, and flow-limiting dissections that often occur during BTK interventions but do so in a manner that avoids the use of a permanent implant. Moreover, by leaving behind a temporary implant, the artery remains unencumbered by a metal stent which may act as an impediment to future interventions and maintains the potential for blood vessel wall remodeling and return of physiological contractility, the holy grail of BTK intervention. However, those theoretical advantages must be evaluated in large-scale clinical trials which compare the Esprit BTK DRS to balloon angioplasty before it is approved for widespread use. Primarily, the LIFE-BTK trial was designed to demonstrate both noninferiority of its safety end point (Freedom from MALE + POD) and the superiority of its efficacy end point (freedom from above-ankle amputation, target vessel occlusion, target lesion binary restenosis and/or CD-TLR) against PTA to support US regulatory approval of this novel device. Second, it will assess a raft of clinically important secondary end points and evaluate the cost-effectiveness of the device in due course. Finally, the PK substudy will gather additional pharmacological safety information, which will be particularly relevant should the study be successful and the device goes on to be utilized in longer, real-world tibial lesions in clinical practice. Enrollment in LIFE-BTK has now been completed, and the release of the initial results is anticipated for the fall of 2023.
